# Coordinated Movement of Cytoplasmic and Transmembrane Domains of RyR1 upon Gating

**DOI:** 10.1371/journal.pbio.1000085

**Published:** 2009-04-14

**Authors:** Montserrat Samsó, Wei Feng, Isaac N Pessah, P. D Allen

**Affiliations:** 1 Division of Anesthesia Research, Department of Anesthesia, Perioperative and Pain Medicine, Brigham and Women's Hospital, Harvard Medical School, Boston, Massachusetts, United States of America; 2 Department of Molecular Biosciences, School of Veterinary Medicine, University of California, Davis, Davis, California, United States of America; University of Texas Austin, United States of America

## Abstract

Ryanodine receptor type 1 (RyR1) produces spatially and temporally defined Ca^2+^ signals in several cell types. How signals received in the cytoplasmic domain are transmitted to the ion gate and how the channel gates are unknown. We used EGTA or neuroactive PCB 95 to stabilize the full closed or open states of RyR1. Single-channel measurements in the presence of FKBP12 indicate that PCB 95 inverts the thermodynamic stability of RyR1 and locks it in a long-lived open state whose unitary current is indistinguishable from the native open state. We analyzed two datasets of 15,625 and 18,527 frozen-hydrated RyR1-FKBP12 particles in the closed and open conformations, respectively, by cryo-electron microscopy. Their corresponding three-dimensional structures at 10.2 Å resolution refine the structure surrounding the ion pathway previously identified in the closed conformation: two right-handed bundles emerging from the putative ion gate (the cytoplasmic “inner branches” and the transmembrane “inner helices”). Furthermore, six of the identifiable transmembrane segments of RyR1 have similar organization to those of the mammalian Kv1.2 potassium channel. Upon gating, the distal cytoplasmic domains move towards the transmembrane domain while the central cytoplasmic domains move away from it, and also away from the 4-fold axis. Along the ion pathway, precise relocation of the inner helices and inner branches results in an approximately 4 Å diameter increase of the ion gate. Whereas the inner helices of the K^+^ channels and of the RyR1 channel cross-correlate best with their corresponding open/closed states, the cytoplasmic inner branches, which are not observed in the K^+^ channels, appear to have at least as important a role as the inner helices for RyR1 gating. We propose a theoretical model whereby the inner helices, the inner branches, and the h1 densities together create an efficient novel gating mechanism for channel opening by relaxing two right-handed bundle structures along a common 4-fold axis.

## Author Summary

Maintaining a precise intracellular calcium concentration is key for cell survival. In skeletal muscle, ryanodine receptor type 1 (RyR1) is an intracellular calcium-release channel that is critical for contraction. Here, we used single-channel techniques to demonstrate the presence of functionally homogenous populations of RyR1 in either the closed or open state and then applied cryo-electron microscopy and image processing to determine the 3D structure of each state. The 3D structures show that RyR1′s ion pathway is formed by two sets of bundles, each containing four rods along a common axis. One set (inner helices) stretches from the lumen to the ion gate, whereas the second (inner branches) stretches from the ion gate to the peripheral cytoplasmic domains. The configuration of the two bundles is clearly different in the two physiological states, allowing a 4 Å increase in diameter of the ion gate upon opening. This diameter increase is sufficient to ensure flow of calcium ions. Upon gating, the cytoplasmic domains undergo a conformational change that converges on the inner branches, revealing a long-range allosteric mechanism that directly connects effectors acting on the cytoplasmic moiety with the ion gate.

## Abstract 3

Three-dimensional cryo-electron microscopy of the RyR1 calcium release channel in the open and closed states reveals its mechanisms of ion gating and long-range allostery.

## Introduction

Maintaining a precise intracellular Ca^2+^ concentration that is 10,000-fold lower than the surrounding environment of the cell, and the ability to dramatically increase intracellular calcium to trigger downstream events in response to specific stimulus are key for cell survival [1]. Ryanodine receptors (RyRs) are high-conductance intracellular Ca^2+^ channels regulated by both exogenous and intracellular mediators, which release Ca^2+^ stored in the endoplasmic reticulum. RyRs are the largest ion channels known, with an average molecular weight of 2.26 MDa, with most of its mass (∼4/5) forming the cytoplasmic domain. The skeletal muscle isoform, RyR1, has a bidirectional interaction with the slow voltage-gated calcium channel in the cell membrane, or dihydropyridine receptor (DHPR), which acts as RyR1′s voltage sensor for cell membrane depolarization [2]. Two key questions to understand RyR1′s function are how are signals transmitted from peripheral cytoplasmic domains to the ion gate, and what is the gating mechanism itself.

Cryo-electron microscopy (cryoEM) and single-particle image analysis of frozen-hydrated RyR1 revealed the 3D structure of RyR1 at approximately 25–30 Å resolution [3–5]. Its cytoplasmic domain is shaped like a flat square prism of 290 Å side and 120 Å high, with at least 12 reproducible domains that have been assigned numerals 1–12 [6,7]. Using the same technique, it has also been possible to map the binding sites for several ligands: the FK506-binding protein 12 kDa (FKBP12), calmodulin (Ca^2+^-CaM, apoCaM), and imperatoxin A (IpTxA) [8–10]. All these interactions, which are known to modulate RyR1 gating, take place at distances at least 130 Å away from RyR1′s putative ion gate and suggest that RyR1 makes use of long-range allosteric pathways between the cytoplasmic sensing domains and the ion gate. The 3D reconstructions of RyR1 in the open conformation indicated several conformational changes involving both the cytoplasmic and transmembrane domains with respect to the closed conformation [11,12]; however, the resolution of these reconstructions (∼30 Å) is insufficient to understand the connection between the two domains or to distinguish the substructure within the transmembrane domain itself.

The wealth of atomic structures of K^+^ channels solved by X-ray crystallography obtained in the last decade has allowed extensive study of the structural rearrangements underlying ion gating for this channel family. In the prevalent model for the ion gating of the K^+^ channel, the inner helices bend outwards around their midpoint (through a Gly or a Pro-X-Pro hinge) to increase the diameter of the ion gate so that it becomes permeable to ion flow. These inner helices are connected to their sensing domains using a plethora of structural arrangements to respond to a variety of effectors (voltage, ion concentration, pH, redox state, small molecules, and ligands). However, with one exception [13], models of K^+^ channel gating have been deduced from comparison of unrelated K^+^ channels. The only other case in which structural data at near-atomic detail is available for both the open and closed states in the same channel is for the nicotinic acetylcholine receptor (nAChR), as determined by electron crystallography [14]. Unlike other K^+^ channels, the nAChR is a pentamer with its ion gate formed by a hydrophobic girdle in the middle of the membrane. Binding of acetylcholine induces a rotation in protein chains that communicates to the inner helices of the pore, resulting in modulation of the ion gate diameter. To date, nothing is known about the ion gating mechanism of RyR1.

Using cryoEM, we previously defined the architecture of RyR1′s transmembrane domain in the closed state at higher detail [7]. RyR1′s closed ion pore is defined by an axial structure formed by two sets of four rods each forming a right-handed bundle, which we defined as the inner helices, and the inner branches. The inner helices shape the core of the transmembrane assembly. The inner branches are in the center of the cytoplasmic assembly and are directly connected with the peripheral cytoplasmic domains. The two bundles converge into a ring of high density, which we presumed to be the ion gate. A second constriction, which would correspond to the selectivity filter, is on the sarcoplasmic reticulum (SR) luminal side of the transmembrane assembly. These two constrictions define a central cavity. The structure formed by RyR1′s inner helices in the closed state appears to be parallel to the canonic structure of the inner helices of closed K^+^ channels [15,16]. A second group of investigators has also reported the presence of the inner helices in the core of the transmembrane assembly of RyR1 in the closed state, achieving similar resolution using the same method and almost identical biochemical conditions [17]. Intriguingly, the conformation that they reported for the inner helices corresponded best to that of an open K^+^ channel, and suggested that the ion gating mechanism used by RyR1 must be radically different than that used by K^+^ channels.

To better understand the basis for RyR1′s gating and to solve the controversy on the conformation of the inner helices in the closed state, we sought to obtain the open and closed conformations of RyR1 in their (frozen) hydrated state using single-particle cryoEM. Furthermore, we used single-channel biophysical characterization of the two states in bilayer lipid membranes (BLMs) using identical samples and conditions, to have a more direct correspondence between conformation and biophysical state of the channel. Here, we present the first demonstration, to our knowledge, of a midlevel resolution 3D model for the open state of RyR1 bound to its accessory protein FKBP12. A 3D reconstruction of RyR1-FKBP12 in the closed state was obtained in parallel for comparison. Thus, in this study, we are able to directly compare both conformations of the same protein, rather than comparing related proteins. Furthermore, both structures correspond to the protein in its fully hydrated state, and both are correlated directly to a functionally characterized biophysical state. We found that upon opening, the cytoplasmic domain undergoes an overall conformational change that involves the connections with the transmembrane domain. In the transmembrane assembly, we find that the inner helices corresponding to the open and closed states of RyR1 have a high cross-correlation with parallel structures of K^+^ channels in the corresponding state. Nevertheless, the ion pathway of RyR1 has features not present in K^+^ channels, which has allowed us to create a novel heuristic model for RyR1′s ion gating.

## Results

### Stabilizing Open and Closed States in RyR1

**Figure 1:**
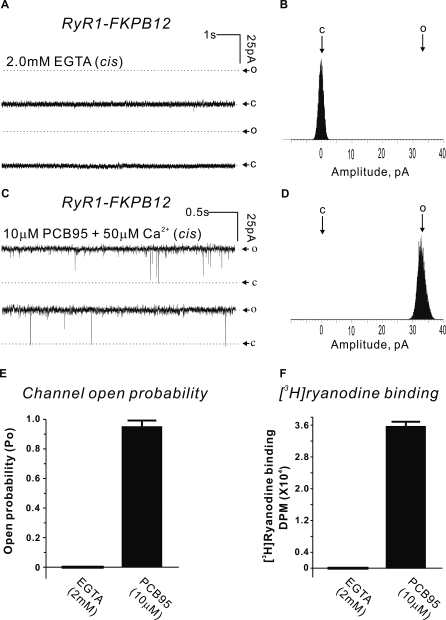
Creating Functionally Homogeneous Open and Closed Datasets of RyR1-FKBP12 (A–E) Purified RyR1 channels were reconstituted in BLMs from a total of four independent junctional SR preparations. Once channels were fused and verified for activity, 2 mM EGTA (pCa^2+^ < 10^8^) perfused into the *cis* chamber produced a fully closed state (C) with no gating transitions to the open state (O) as shown by the BLM traces and amplitude histogram of the representative channel (A and B). This behavior was observed in four of four channel reconstitutions (E). Pretreatment with PCB 95 (10 μM) persistently stabilized the full open state of RyR1 as shown by the representative channel (C and D). The *cis* solution contained 50 μM Ca^2+^, 10 μM PCB 95, and 100 nM FKBP12 throughout the recording. This behavior was observed in ten of ten reconstitutions (E) and lasted the duration of each recording (ranging between 12 s and 6 min). (F) [^3^H]ryanodine binding to RyR1 junctional SR vesicles in the presence of 2 mM EGTA show negligible specific high-affinity binding or in the presence of 50 μM free Ca^2+^ and 10 μM PCB 95 shows >3.5 × 10^4^ dpm specific binding. The data summarize results from four independent measurements obtained from two different junctional SR preparations.

To obtain the resolution necessary for the visualization of secondary structure (∼9 Å), it is critical to obtain a highly homogeneous dataset. Obtaining a homogeneous population of RyR1 in the closed state is relatively easy. By contrast, the typical flickering behavior of RyR1 under physiologic activating conditions represents a significant limitation, since it produces a mixed population of open and closed states, e.g., under maximum Ca^2+^ activating conditions (50 μM Ca^2+^ on the cytoplasmic side), the channel open probability (*P*_o_) of reconstituted purified RyR1-FKBP12 channels is less than 30% (unpublished data). Our previous studies using vesicles demonstrated that the neuroactive noncoplanar polychlorinated biphenyl 2,2′,3,5′,6-pentachlorobiphenyl (PCB 95) had an unprecedented activating effect on RyR1 [18,19], suggesting that it would be a candidate small molecule to stabilize RyR1′s open state. The BLM studies of reconstituted purified RyR1-FKBP12 channels indicate that PCB 95 stabilizes the full open (conducting) state in ten out of ten reconstituted channels, resulting in extremely long-lived openings interspersed with rare short-lived transitions to the closed state. This results in a mean *P*_o_ of 0.96 and thus produces a highly homogeneous dataset (Figure 1C–1E). By contrast, addition of 2 mM EGTA to the *cis* chamber (pCa^2+^ < 10^8^) after fusion of an actively gating channel completely stabilized the fully closed state of the channel with no gating transitions observed for the entire recording period (*P*_o_ = 0) (Figure 1A, 1B, and 1E). High-affinity [^3^H]ryanodine binding experiments query the conformational state of a large number of RyR1s [20]. The presence of EGTA (2 mM) in the assay buffer negates specific binding of [^3^H]ryanodine because the channels are in a closed conformation. By contrast, the presence of PCB 95 and optimal Ca^2+^ produced nearly 10 pmol of binding sites per milligram of SR protein (∼35,000 disintegrations per minute [dpm]/25 μM of SR protein) at steady state (Figure 1F), indicative of the fact that the channels are stabilized in the open state. These biophysical and biochemical data provide two independent measures of the ability of PCB 95 to stabilize the open state of the RyR1 channel having a unitary current level indistinguishable from a native channel in the full open state. The unitary current is a fundamental parameter for any given channel [21], thus it is safe to assume that the PCB 95–stabilized RyR1 has a pore structure representative of the native open state (in which only the kinetic/thermodynamic parameters have been altered). To exert its effect, PCB 95 requires that RyR1′s FKBP12 accessory subunit be bound [22]. In vivo, FKBP12 is constitutively bound to RyR1 and is known to stabilize its fully closed state and minimize subconductance states [23,24]. Both the position and orientation of FKBP12′s atomic coordinates with respect to RyR1 have been mapped and have been shown not to alter RyR1′s closed-state conformation at 16 Å resolution [9].

### Cryo-Electron Microscopy of RyR1

**Figure 2:**
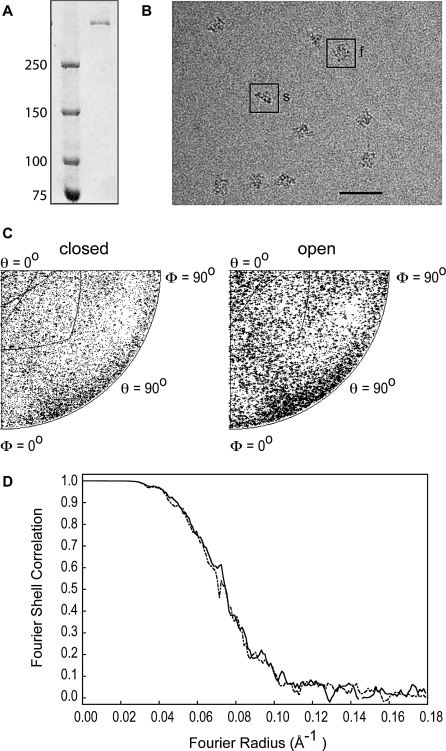
Biochemistry, Cryo-Electron Microscopy, and Single-Particle Image Processing (A) SDS-PAGE of purified RyR1 (right lane) with molecular weight markers on the left. (B) Electron microscopy of a field of RyR1 particles embedded in ice, with the particles showing clear substructure. A 4-fold view and a side view are highlighted with the letters f and s, respectively. Scale bar indicates 100 nm. (C) Plots of the angular distributions of the particles used in the 3D reconstructions in the closed and open conformations, showing a uniform distribution of Euler angular orientations of the vitrified RyR1. (D) The Fourier shell correlation curves indicate 10.2 Å resolution for the closed (continuous line) and open (dashed line) datasets according to the 0.143 cutoff criterion.

Our RyR1 purification method [7] produced a single band on PAGE (Figure 2A) indicative of a pure RyR1 preparation, and RyR1s with well-preserved structure when viewed with cryoEM (Figure 2B). The relatively high concentration of RyR1, approximately 2 mg/ml, enabled the successful cryo-preparation of RyR1 suspended over holes instead of lying on a carbon support, a method that allows increased resolution of the 3D reconstruction because it considerably increases the randomness of orientations [7]. CryoEM and image processing of two frozen-hydrated RyR1-FKBP12 datasets corresponding to the open and closed states, with approximately 17,000 particles each, yielded two 3D reconstructions. The homogeneous angular distribution for both datasets (Figure 2C) indicates that all orientations are equally represented in both datasets; thus the two 3D reconstructions have isotropic resolution and are free of the missing-cone artifact [25]. The nominal resolution of the two reconstructions, 10.2 Å, was determined by Fourier shell correlation (FSC) using a cutoff criterion of 0.143 [26] (Figure 2D), which in this study was a conservative value relative to the five times noise-correlation cutoff. The resolution value of 10.2 Å appears reasonable, taking into account the fact that in general, positive identification of secondary structure is indicative of 9 Å or better resolution. We have focused our analysis on only those structures readily visible in the cryoEM density map without any further manipulation. Specifically, we have centered our study on structures with densities at least 2.8 σ levels above the mean value.

### Ion Gate Opening Mechanism: Conformational Changes in the Cytoplasmic Assembly

**Figure 3:**
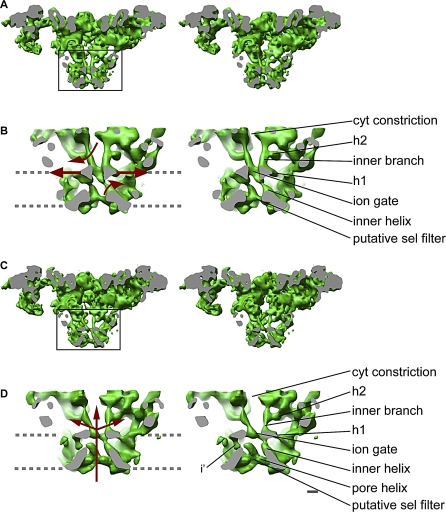
Stereo View of RyR1 in the Closed and Open States (A and C) Side view of RyR1 in the closed and open states, respectively, sliced through the 4-fold axis, along a plane that is 11° from the diagonal, with the region indicated with a square magnified in (B and D). The cutting plane is in gray. The density maps are displayed at a higher threshold to reveal the inner helices and the inner branches. Note the less crowded area between the inner branches. (B and D) The structure surrounding the ion pathway in the closed and open conformations, respectively. The region enclosed in the square in (A and C) has been magnified, and a thin slice has been cut from the back using another cutting plane parallel to the front one for better visualization of the ion pathway. The two bundles formed by the inner branches and the inner helices define the ion gate at their meeting point. Because the bundles are cut through the middle and they move upon gating, in the closed state, two inner helices are seen, whereas in the open state, one helix is seen fully and two others are partially sliced. Two inner branches are seen fully both in the open and closed states. The arrows in (B) indicate how the different structures move upon opening. Upon channel opening, the inner helices bend outwards, presumably on a Gly hinge in the peptide sequence of the presumed inner helix, the inner branches move apart, and the horizontal structures h1 move outwards. Their combined effect results in dramatic changes along the ion pathway and produce a potential novel mechanism for Ca^2+^ channel gating. In (D), the arrows indicate the pathway of the ion flow. The identifiable features are indicated, and the abbreviations are as follows: cytosolic constriction (cyt constriction), horizontal rod-like structures (h1, h2), possible continuation of the inner helices (i'), and putative selectivity filter (putative sel filter). The gray dashed lines indicate the approximate boundary of the SR membrane. Scale bar indicates 10 Å.

**Figure 4:**
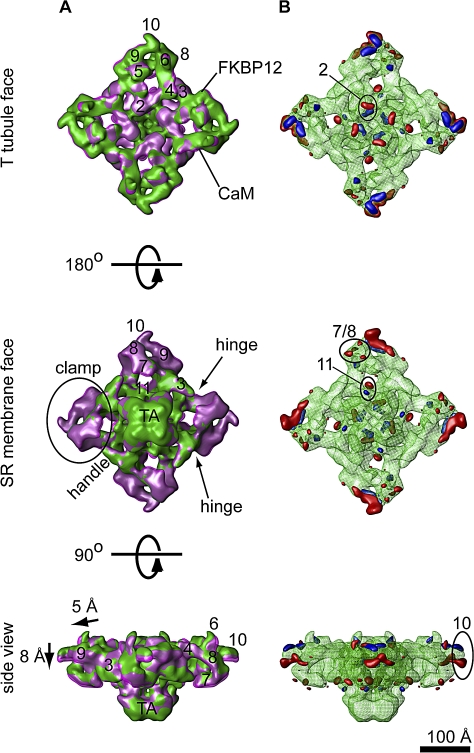
Domain Translocation upon Opening (A) The superimposition of RyR1′s isosurfaces in the open (magenta) and closed (green) states reveals a generalized conformational change. Upon opening the clamp, domains (especially the distal domains 7, 8, and 10) move toward the SR, domain 2 moves upwards (towards the T-tubule) and outwards, domain 11 moves outwards, and the transmembrane assembly rotates clockwise with respect to the cytoplasmic domain. The orientation of each view is indicated on the left, and the relevant features are circled. TA, transmembrane assembly. (B) The two difference maps corresponding to the extra mass in the open (red) and closed (blue) states are superimposed on the 3D reconstruction of RyR1 in the closed state (green mesh). The “pairs” of neighboring differences correlate with the main changes observed in (A). The volumes have been filtered to a resolution of 18 Å for better visualization of the main conformational changes. The approximate binding sites of Ca^2+^-CaM and FKBP12 are indicated. IpTxa binds at a position overlapping the Ca^2+^-CaM binding site.

When comparing the 3D reconstructions corresponding to closed and open RyR1, they look rather similar (Figure 3). However, careful analysis reveals that they are different conformomers of the same molecule. The coarse conformational changes may be better appreciated when the two 3D reconstructions are filtered to lower resolution and directly superimposed (Figure 4A), or when the two 3D structures alternate between the closed and open states (Video S1). Whereas most of the domains appear to move, the largest conformational changes take place in the distal regions of the cytoplasmic domains. The larger conformational changes are also evident in the 3D difference maps (Figure 4B). The difference was performed in both directions (closed minus open, and open minus closed), providing the regions of mass that were exclusive for the closed and open states, respectively.

Because the open- and closed-state datasets were processed in parallel, starting from a common low-resolution structure, and result in clearly different conformations, we believe that these are genuine representations of the two physiological states. Furthermore, given the large dimensions of the RyR1 (e.g., 30× larger than the K^+^ channel KcsA), domains separated by more than 100 Å may be regarded as resolved independently from each other. Yet, these domains follow the same direction of movement when they are connected by intervening density. Finally, for each domain that moved, there is a pair of complementary differences (see Figure 4B), which is also indicative of high data quality and actual movement.

The largest-magnitude conformational change occurs in the cytoplasmic domain, whereby each of the quadrants swivels outwards. The corners or clamp domains (domains 9 and 10) together with the structure formed by domains 7, 8, and 8a move away from the T-tubule and towards the SR membrane by approximately 8 Å. Concomitantly, domain 2, more central and facing the T-tubule, moves approximately 4 Å towards the T-tubule, and outwards away from the 4-fold axis (Figure 4A). We do not see an opening of the clamp domains in the open state as was suggested previously (see Discussion). Domain 6, protruding towards the T-tubule, moves approximately 5 Å outwards when the channel is in the open state, and a similar magnitude of outward movement takes place at domain 11, facing the SR membrane. The main effect of this swiveling movement is that the mass moves from the center to the outside, making the 4-fold axis less crowded. This movement in the cytoplasmic regions is clearly conveyed to the inner branches (Figure 3A and 3C, Video S1).

### A Reproducible Model of RyR1′s Ion Pathway

**Figure 5:**
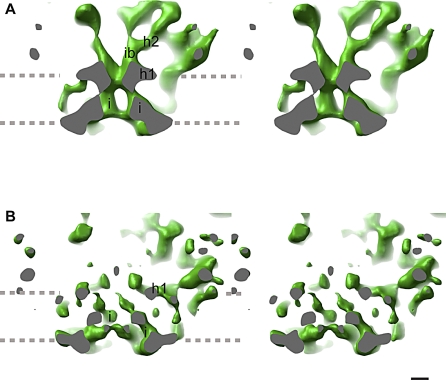
Previously Determined Closed RyR1 Ion Pathways (A) RyR1 in the closed state by Samso et al., 2005 [7] (EMDB code 5014). (B) RyR1 in the closed state by Ludtke et al., 2005 [17] (EMDB code 1275). The orientation and representation of both 3D reconstructions is equivalent to that of the RyR1-FKBP12 3D reconstructions in the open and closed states shown in Figure 3. The thresholds of the two compared structures have been adjusted for optimal comparison. The identifiable features are indicated as follows: inner helices (i), inner branches (ib), and horizontal rod-like structures (h1). The gray dashed lines indicate the approximate boundary of the SR membrane. Scale bar indicates 10 Å.

**Figure 6:**
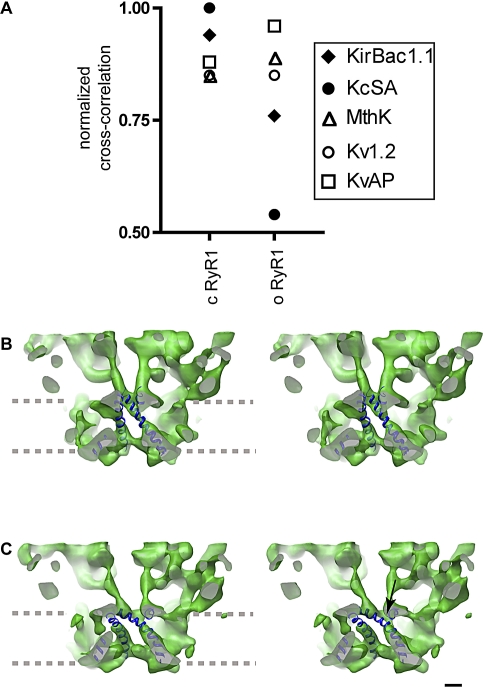
RyR1 Inner Helices in the Context of the Inner Helices of K^+^ Channels (A) Cross-correlation between the inner helices of five K^+^ channels and these of RyR1 measured for both the closed and the open RyR1. The filled symbols are indicative of K^+^ channels in the closed conformation, and the open symbols are indicative of those in the open conformation. Closed K^+^ channels cross-correlate better than open K^+^ channels with the closed RyR1, and the situation is reversed for the open state. (B) Stereo pair showing the docking of the inner helices of closed RyR1 with these of closed KcsA. The gray dashed lines indicate the approximate boundary of the SR membrane. (C) Stereo pair showing the docking of the inner helices of open RyR with these of open KvAP. The arrow points to the density discontinuity in the inner helices of RyR1 that overlaps with the Gly hinge in the docked K^+^ channel. Scale bar indicates 10 Å.

**Figure 7:**
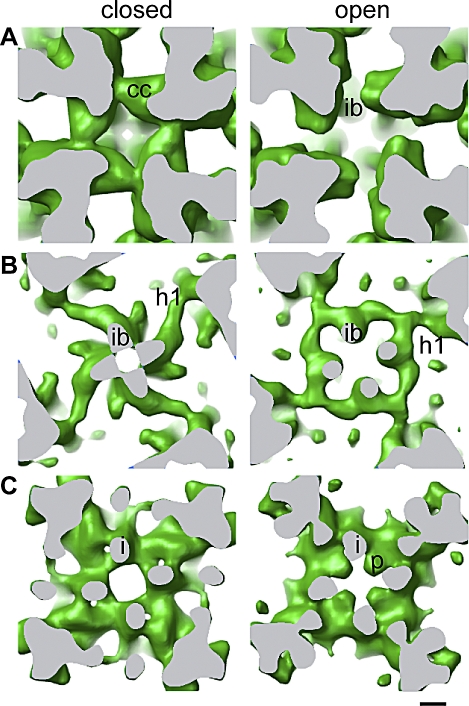
Isosurface Representations Showing the Main Constrictions along the Ion Pathway in the Closed and Open States (A) The cytosolic constriction relaxes and the inner branches become more separated in the open state. (B) The ion gate increases in diameter upon opening. (C) The pore helices are identifiable in the open state. The identifiable features are indicated as follows: cytosolic constriction (cc), inner helices (i), inner branches (ib), pore helices (p), and horizontal rod-like structure (h1). The region between the ion gate and the cytosolic constriction is continuous with the cytoplasm through the fenestrations situated between the inner branches. The cutting surface plane is gray in all panels, and the structures are viewed from the cytosolic side. The isosurface level for panels (B and C) is equivalent to that in Figure 3. The isosurface level for (A) has been lowered in order to display the cytosolic constriction. Note that while the ion gate and the cytosolic constriction are wider in the open state, the selectivity filter appears narrower. Scale bar indicates 10 Å.

**Figure 8:**
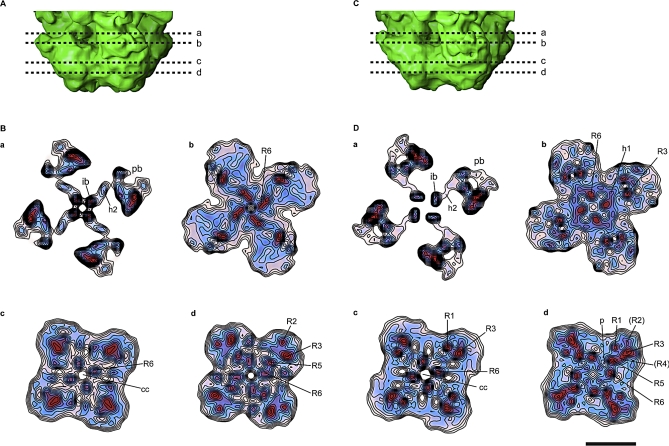
Slices through the Transmembrane Domain of RyR1. (A) Side view of the transmembrane domain of RyR1 in the closed state with the positions of the slices indicated with dashed lines. (B) Slices of the RyR1 in the closed state perpendicular to the 4-fold axis as seen from the cytoplasmic domain. The blue and red colors indicate a density of at least 2.35 σ and 3.06 σ above the mean, respectively. The contour intervals correspond to 0.176 σ increments. The higher gradient of density surrounding the perimeter of the transmembrane domain indicates the boundary of the structure. The numerals preceded by the letter R indicate putative RyR1 α helices that correspond to the six transmembrane α helices of Kv1.2 (see Figure 9); R6 corresponds to what we have previously defined as the inner helices. The numerals in parentheses indicate less defined densities that correspond in position to α helices in Kv1.2. The abbreviations are as follows: cc, central cavity; h, horizontal densities; ib, inner branches; ih, inner helices; p, pore helices; and pb, peripheral branches. (C) Side view of the transmembrane domain of RyR1 in the open state with the positions of the slices indicated with dashed lines. (D) Slices of the RyR1 in the open state perpendicular to the 4-fold axis as seen from the cytoplasmic domain and displayed as in (B). Scale bar indicates 50 Å.

In the closed state, the overall structure of the inner helices and inner branches of RyR1-FKBP12 display a structure almost identical to the structure of the closed state of RyR1 that we determined previously in the absence of FKBP12 [7] (compare Figures 3B, 3D, and 5A). As in our earlier report, the inner helices have a tepee-like arrangement that overlaps directly with the tepee structure described for the ion pathway in the atomic models of K^+^ channels [15,16,27–29] (e.g., see Figure 6). Although a resolution of 9 Å or better is needed to visualize α helices [30,31], it has been described that resolution of 10 Å or even less may suffice to identify α helices, if they are separated from surrounding structures [32]. Another report of the closed state of RyR1 at 10 Å [17] also indicated four inner helices in the same location—although in a different configuration—supporting this finding (Figure 5B). When compared to our closed-state reconstruction, the inner branches in the open state are clearly recognizable but in a different conformation, and the central passage has significantly lower density than in the closed state (stereo pairs shown in Figures 3B and 5A). The inner branches and inner helices define three main constrictions along the 4-fold axis, represented in Figure 7. The upper, or cytosolic, constriction is defined by the distal enlargement of the inner branches (Figure 7A). The meeting point between the inner branches and the inner helices defines the ion gate (Figure 7B). The lowest constriction defines the opening to the SR lumen (Figure 7C), and is formed by the pore helices in a region that corresponds to the selectivity filter of the K^+^ channels. The inner helices, the ion gate, and the putative selectivity filter surround the central cavity (see Figures 3, 8B-c, and 8D-c).

**Figure 9:**
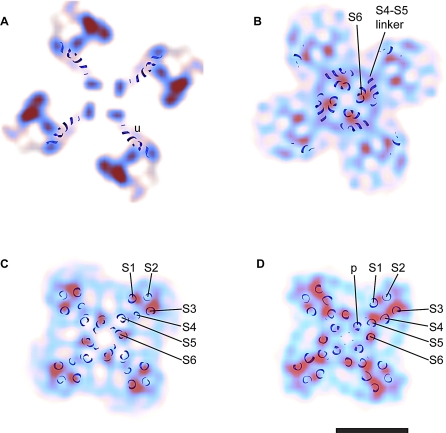
Slices through the Transmembrane Domain of Open RyR1 Superimposed on the Atomic Structure of Open Kv1.2 (A–D) Slices through the transmembrane domain of RyR1 in the open state perpendicular to the 4-fold axis corresponding to the positions a–d indicated in Figure 8C. The blue and red indicate a σ of at least 2.35 and 3.06 above the mean, respectively, with the slices b–d in lighter color to more clearly visualize the docked α helices of the K^+^ channel. The atomic model of Kv1.2 is superimposed; only the α helical backbone is displayed. S1–S6 are transmembrane segments, p indicates the pore helix, and u an α helix within the atomic model of Kv1.2 of unknown sequence. Scale bar indicates 50 Å.

In agreement with our previous 3D structure of RyR1 [7], the transmembrane assembly of both new 3D reconstructions reveals at least six distinct regions of high density per subunit that can be attributed to α helices (Figure 8). These rod-like structures have a density >3 σ above the mean and are clearly differentiated from their surroundings; they are identified as red contoured regions in Figure 8. There is a remarkable similarity between the arrangement of all six α helices of the mammalian voltage-dependent shaker channel Kv1.2 in the open state and the putative RyR1 transmembrane α helices in the same condition (see Results and Figure 9). For the purpose of comparison, we designate the putative α helices of RyR1 as R1–R6, where R6 is the inner helix. These are named according to the comparably positioned α helices of the K^+^ channel (S1–S6).

### Ion Gate Opening Mechanism: Dissecting the Ion Pathway

#### Movement of the inner helices away from the 4-fold axis.

In the closed state, the inner helices form a right-handed helical bundle constricting towards the cytoplasm (Figure 3). The length of the inner helices is approximately 40 Å (from lumen to ion gate). A slight decrease in density in the region closest to the cytoplasmic side of the membrane indicates a point of flexibility. In the open conformation, the bottom halves of the inner helices (proximal to the lumen) are reoriented in four ways with respect to the closed state. They are shifted 3 Å along the 4-fold axis towards the cytosol, they are in a more vertical position with respect to the 4-fold axis—39° versus 44°—causing the portion facing the ion gate to move farther away from each other, and they are slightly bent outwards (Figure 3D). After this bend, the electron density of the inner helices becomes very weak. The inner helices clearly point to a ring of density that has a larger diameter and is less thick in the open state than in the closed state (Figures 3B, 3D, and 7B). These data confirm our previous assumption that this ring of density is the ion gate. Both the change in angle and the outwards bending appear to be directly related to a relaxation of the helical bundle and the wider ion gate. The length of the inner helices in open conformation is approximately 20 Å (from lumen until the density discontinuity). There are two equally likely possibilities for the pathway of the inner helix beyond the region of discontinuity: (1) the inner helix of RyR1 directly connects with the ion gate at the base of the adjacent inner branch, or (2) the inner helix connects with the ion gate via a short rod-like density connected to the ion gate that appears in the open state indicated by i′ in Figure 3D. Because of its more contorted trajectory, the second possibility may require an additional hinge, which would be a viable possibility based on our putative sequence assignment of the inner helix presented below.

To assess the similarity between the conformation of the inner helices of RyR1 and K^+^ channel families, we compared this region of the channel to K^+^ channels by docking the inner helices of K^+^ channels into the cryoEM density map, followed by computation of the cross-correlation values between the two maps filtered at 10 Å resolution. We considered all the currently deposited atomic structures for the K^+^ channels that include K^+^ channels crystallized in the closed (KcsA and KirBac1.1) and the open (Kv1.2, KvAP, and MthK) states [15,16,27,33,34]. Pairwise comparisons indicate that the inner helices of closed-state RyR1 cross-correlate better with all closed K^+^ channels than with any of the open K^+^ channels (Figure 6A, filled symbols). The opposite is true for the inner helices of the open-state RyR1 (Figure 6A, open symbols). The best matches are with the closed KcsA and open KvAP for the closed and open RyR1s, respectively; see stereo pairs for each 3D reconstruction with the docked K^+^ channels (Figure 6B and 6C). In the docking, the region of density discontinuity observed in the open cryoEM maps directly superimposes with the Gly (or the equivalent Pro-X-Pro) hinge of the K^+^ channels [28,29,35] (arrow in Figure 6C), supporting the hypothesis that this is the site of the putative Gly hinge of RyR1 [36].

**Figure 10:**
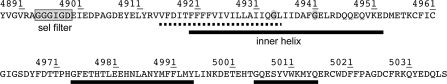
Secondary Structure Prediction Predicted regions of hydropathy (dashed line) and α helix (continuous line) for the C-terminal region of RyR1. The position of the selectivity (sel) filter is also indicated. The Gly residues (highlighted in gray) are possible points of flexibility.

On the basis of hydropathy profiles, early studies suggested that amino acids (AA) from 4914 to 4937 make up the last transmembrane domain of RyR1 [37], and it has also been proposed that the last transmembrane domain corresponds to the inner helix of the channel [38,39]. This putative transmembrane domain has only a single Gly residue near its C-terminus, 4934. However, the position of this residue is too close to the end of the helix to act as an effective hinge. For a hinge to change the diameter of the ion gate effectively, it would need to be located in a more central location. Exclusively on the basis of structural data, others have proposed that instead, AA 4918–4948 [17] defines the last transmembrane domain. We favor this assignation. The reason for this preference is based on the fact that unlike the hydropathy profiles, α helix prediction algorithms indicate a low α helical propensity for sequence 4911–4919, and high α helical propensity for residues 4920–4952 (Figure 10). Furthermore, in the latter inner helix assignments, Gly 4934 is in a more central position, and a second Gly residue, AA 4941, could add a second point of flexibility within the inner helix. This assignment would also place Phe 4940 in a position where it could act as a putative pore-blocking residue in the closed state [16,40]. In addition, Asp 4938 and 4945, two negative residues known to modulate RyR1 ion fluxes [41], would be located after the Gly 4934 hinge and thus be in the cytosolic vestibule, affording them the opportunity to efficiently perform such a function. Last, the longer proposed sequence (residues 4920–4952) agrees better with the 40 Å length that we measure for the complete inner helix.

#### Movement of the inner branches away from the 4-fold axis.

In our previous closed-state 3D reconstruction, we identified four rod-like structures situated near the 4-fold axis of the structure on the cytoplasmic side of the presumed ion gate, that we named inner branches [7]. The distinct section of the inner branches in our two new 3D reconstructions, with densities >3 σ above the mean value, is approximately 21 Å long with an elongated lateral extension in the middle (h2) (Figures 3, 8B-a, and 8D-a). Both the inner branches and their lateral extension then merge with other internal cytoplasmic domains.

In the closed state, the inner branches merge with each other and form the high-density ring defining the ion gate. In the transition from the closed to the open states, the four inner branches assume a more tilted position (from ∼21° to 29° with respect to the 4-fold axis), rotate 5° counterclockwise (as seen from the cytoplasmic distal domains), and move approximately 6 Å further away from each other at their midpoint. The separation of the inner branches on the cytoplasmic side of the SR membrane is directly related to the diameter of the ion gate (Figures 3, 7A, 7B, 8B-a, and 8D-a). Unlike their position in the closed state, in the open state, the inner branches do not merge, but are still attached to the wider ring (Figures 3, 7A, and 7B). Thus, it appears that by changing their position during the transition from closed to open, the inner branches participate directly in the change of the diameter of the ion gate. By directly linking the peripheral cytoplasmic domains (where many RyR1 effectors interact) to the ion gate, the inner branches appear to be strategically built to mediate long-range conformational changes, as we suggested previously based on the closed-state structure alone.

The inner branches become bulkier approximately 25 Å away from the ion gate, and in the closed conformation, they contact each other forming a 10 Å–diameter ring (the cytosolic constriction) (Figures 3 and 7A). The cytosolic constriction does not appear to have a direct role in gating since the cavity between the four branches is continuous with the cytosol through long openings similar to the side portals identified in the nAChR [42]. In the latter case, the side portals are thought to perform an electrostatic prescreen of ions. In RyR1, in which this structure is situated after the putative ion gate, we do not expect such a functional role. However, it is conceivable that the cytosolic constriction has a role in stabilizing the closed conformation.

The sequence identity of the inner branches is not known. Since they are a density continuous with that of the ion gate, one possibility is that they are formed by part of the 4938-5037 C-terminal sequence downstream of the inner helices. We base this on the following reasoning. First, this sequence is predicted to contain critical residues involved in multimerization [43], thus a position near the 4-fold axis is a likely location because the ion gate, and/or the cytosolic constriction 25 Å away from the ion gate, would be a good candidate(s) as a multimerization site(s). Second, one of the predicted α helical segments of this region (Figure 10) has an estimated length of 21 residues, which is compatible with its physical measurements of 25 Å. Third, based on electron paramagnetic resonance (EPR) studies of the full-length KcsA channel, the sequence downstream of the inner helices was proposed to be formed by a mixture of α helix and random coil with a combined length of approximately 40 Å and a structural arrangement very similar to that found in our studies, whereby the four elongated structures run approximately parallel to the channel's 4-fold axis extending towards the cytoplasm [44]. This region has not been resolved in any of the K^+^ channel atomic models determined by crystallography that we have checked [15,16,27–29]. However, the complete structure of the inner branches must be more complicated than a simple combination of α helix and random coil since the rod-like density h2 emerges from each inner branch at a 90° angle and merges with the cytoplasmic domains (Figures 3, 8B-a, and 8D-a).

#### Outward movement of a planar network surrounding the ion gate.

Four additional structures parallel to the cytosolic side of the SR membrane connect the ring forming the putative ion gate to the peripheral region of the transmembrane domain (h1, see Figures 3 and 7B). In the closed state in which the ion gate is smaller, the four rod-like h1 structures give the appearance of a cross. A similar structure in the K^+^ channel, the slide helix, is also proposed to have an active role in gating [7,17]. The h1 densities appear to move outwards in going from the closed to the open state, also contributing to the increased diameter of the ion gate in the open state. The outward movement of the h1 densities is concomitant with the outward and upward (toward the cytoplasm) movement of the inner helices upon opening (arrows in Figure 3B), which accounts for the slightly wider and shorter appearance of the transmembrane domain in the open state.

#### Pore helices.

Cation selectivity is performed at the selectivity filter, which has been shown to have a conserved sequence among several different cation channels such as K^+^ channels, IP3Rs, and RyRs [40,45]. Single point mutations in the identical six-residue sequence of all RyRs (GGGIGD) can dramatically reduce the unitary conductance or even abolish it [46–48]. In all the crystallized K^+^ channels, the selectivity filter forms a constriction that spans from the tips of the pore helices to the pore opening opposite to the ion gate. There the carbonyl residues coordinate the cations along their pathway. In our 3D reconstructions, a similar constriction between the central cavity and the luminal mouth constitutes the putative RyR1 selectivity filter. In the open state, the pore helices are clearly resolved. They protrude into the central cavity, providing a narrow passage between the central cavity and the SR lumen (Figures 3D and 7C). We are certain that pore helices in the presence of PCB 95 reflect the native open conformation of the permeation pathway because the PCB 95–stabilized conformation has a unitary Cs^+^ current indistinguishable from that measured in the presence of activating Ca^2+^ alone (Figure 1C). In the closed state, either in the absence [7] or in the presence of FKBP12, the density of the pore helices is not resolved (Figures 3B and 7C). This could be due to lack of resolution per se, or alternatively, they are closer to another density than in the open state. This can only be solved when higher resolution is attained for both states.

The change in conformation along the ion pathway during gating can be seen in Video S2.

### Putting the Ion Gate Dimensions into Context

**Figure 11:**
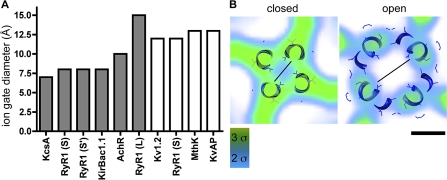
Ion Gate Diameters of K^+^ and RyR1 Channels (A) Comparative chart of ion gate diameters. The filled and open bars indicate closed and open channels, respectively. Three diameters are reported for closed RyR1: (S) from the current RyR1-FKBP12 reconstruction, (S′) from our previous RyR1 reconstruction [7], and (L) from a previous RyR1 reconstruction done by another group [17]. (B) Section across the ion gate of RyR1 in open and closed states with the docked K^+^ channels in the corresponding conformations. The black lines measure the distance between the inner edges of the inner helices of the K^+^ channels and the corresponding densities within RyR1. Scale bar indicates 10 Å.

We compared the diameter of the ion gate of our open/closed RyR1-FKBP12 with that of previous 3D determinations of RyR1 in the closed state [7,17]. Furthermore, taking into account that the diameter of the K^+^ and Ca^2+^ ions is very similar, around 4 Å, we compared the diameter of the ion gate of RyR1 with that of the K^+^ channels and nAChR in open/closed conformations that have been determined at atomic resolution. To accomplish this for RyR1, we measured the diameter of the ion gate at a density threshold corresponding to the secondary structure (Figure 11). The diameter of the ion gate of our closed RyR1-FKBP12 and closed RyR1 [7] 3D reconstructions is 8 Å, whereas the diameter of the ion gate of the closed RyR1 obtained at similar resolution in the same conditions by another group is 15 Å [17]. The diameter we find here for the RyR1-FKBP12 ion gate in the open state is 12 Å. From the known atomic structures of K^+^ channels and nAChR, we took the equivalent measurement, defined by the inner edge of the inner helices, and find that in the closed state, their ion gate diameters range between 7–8 Å (closed K^+^ channels, which are tetramers) and 10 Å (closed nAChR, which is a pentamer). For all of the open channels, the diameter is 12–13 Å [29,33–35].

Taken into this general context, our measurement of 8 Å for RyR1′s closed ion gate falls within the values found for the closed conformations, and the measurement of 12 Å for RyR1′s open ion gate corresponds to that found for the open conformations of the known K^+^ channels (Figure 11). When the side chains of the K^+^ channel's atomic model are taken into consideration, the actual diameter of the closed pore is 4 Å [15]. Thus, it is likely that when atomic resolution of RyR1′s structure is obtained, our 8 Å diameter will result in similar pore dimensions, which is an appropriate conformation for a closed Ca^2+^ channel. Likewise, the observed increase to 12 Å diameter in the open state should be sufficient to enable Ca^2+^ flow.

### Similarity of the Architecture of the Transmembrane Domains of RyR1 and Kv1.2

When the atomic structure of open Kv1.2 [35] is superimposed on the open RyR1 density map, the positions of the α helices of Kv1.2, S1–S6, correlate well with high-density regions of RyR1 (Figure 9). Starting from the 4-fold axis, we assign S6, the four inner α helices of the K^+^ channel, to the four central rod-like structures (inner helices) in RyR1 (R6, see Figures 8D-b through 8D-d and 9B–9D). The tips of the pore helices in Kv1.2 also overlay those of RyR1 (p in Figures 8D-d and 9D). Four rod-like structures that are in the same position as the outer helices of the K^+^ channel (S5) can be identified in the region of RyR1′s transmembrane domain proximal to the lumen (R5) (see Figures 8B-d, 8D-d, and 9D). We suggest that they are the putative outer helices, or R5. The S1–S4 helices form the voltage sensor of Kv1.2. Although RyR1 does not have known voltage-sensing activity, we observe that S1–S4, which form the voltage sensor in Kv1.2, overlap with the corners of the transmembrane assembly of RyR1. Two densities in RyR1, R1 and R3, are in a similar configuration to S1 and S3, although slightly farther away from the 4-fold axis (Figures 8D-c, 8D-d, 9B, and 9D). R2, a weaker density, matches with S2, and the intervening density between R3 and R5, indicated as R4 in Figure 8D-d, could correspond to S4. At the level of the ion gate, R6 continues to overlap with S6, and the horizontal rod-like density 1 (h1) of RyR1 overlaps with the S4–S5 linker structure (Figures 7B, 8D-b, and 9B). The h2 structure coincides with an α helical structure of unknown sequence in the Kv1.2 atomic model [35] (see u in Figure 9A superimposed on the open RyR1 density map). Despite the structural similarity, we could not find sequence homology between the transmembrane segments of Kv1.2 and the aliphatic segments of RyR1. In contrast with Kv1.2, two other atomic models of K^+^ channels with six transmembrane α helices per subunit [29,34] do not match well with our cryoEM density map. The region of discordance in these atomic models is the S1–S4 formation; however, this could well be the result of the presence of the Fab/Fv fragments against the voltage sensors that were needed for crystallization.

## Discussion

### Comparison with Previous Open-State 3D Reconstructions of RyR1

A previous 30 Å resolution reconstruction of RyR1 was prepared in conditions designed to represent the open conformation (100 μM Ca^2+^, 100 nM ryanodine) [11]. This reconstruction indicated that in going from the closed to the open state, the protein undergoes several conformational changes: a counterclockwise rotation of the transmembrane domain with respect to the cytoplasmic assembly, an elongation of approximately 10 Å of the overall structure in the 4-fold axis direction, an opening of the clamp domains between domains 9 and 10, and an increase in pore diameter from 0 to approximately 18 Å. Two other 3D reconstructions of RyR1 at similar resolution were prepared to represent fully and transiently open states (100 μM Ca^2+^, and 100 μM Ca^2+^ plus 1 mM AMP-PCP, respectively) [12] also indicated an opening of the clamp domains between domains 9 and 10. In these cases, no elongation along the 4-fold axis was observed. Due to the limited resolution, the pore diameter was highly threshold-dependent and in a range between 0–7 Å diameter.

Many of these features are not compatible with our observations, and because of the low resolution of these reconstructions, some genuine structural differences were likely to have been confounded by effects resulting from low or anisotropic resolution. In our current reconstructions and a previous closed-state reconstruction [7] of RyR1, all at around 10 Å resolution, the only connection from domain 10 to the rest of the structure is domain 9, making it impossible for the clamp domains to “open” during gating by separating domains 10 and 9 [11,12]. Their observed gap is likely to be a consequence of the lower resolution and the threshold nonequivalence between the open state and closed states as it is known that the choice of threshold in low-resolution reconstructions dramatically affects the surface representations. Second, the elongation in the *z* direction that they observed, but was not observed in our reconstruction, is likely due to averaging of the central domains moving away from the transmembrane assembly and the peripheral domains moving toward it. In addition, the missing-cone artifact [25], whereby a large proportion of 4-fold views with respect to side views, could provoke an artifactual elongation along the 4-fold axis. Last, the diversity of dimensions for the open pore and the much lower resolution in the previous open-state 3D reconstructions [11,12] do not warrant a comparison of pore dimensions.

### Control of the Ion Gate from Remote Cytoplasmic Domains

In trying to elucidate the molecular mechanism for ion gating using cryoEM, we have found that upon channel opening, structural changes in the cytoplasmic domains are coordinated with structural changes in the ion gate. All domains appear to move in an orchestrated manner, resulting in a significant lowering of density along the 4-fold axis of the protein and an increase of the ion gate diameter. The most obvious connection that we can see between changes at the cytoplasmic domains and ion gate opening is how the upward and outwards movement of the cytoplasmic domains pulls the inner branches in that same direction. Because the inner branches are directly connected to the ion gate, it is straightforward to see how their being pulled apart increases the diameter of the ion gate.

RyR1′s large cytoplasmic domain interacts with several proteins such as the voltage sensor (DHPR), FKBP12, CaM, and IpTxa, and all four affect RyR1′s gating. In intact skeletal muscle, RyR1 appears to open exclusively under the control of the DHPR. Removal of FKBP12 or addition of IpTxa is known to induce subconductance states, whereas CaM modulates the Ca^2+^ dependence of RyR1′s probability to open. The binding sites for FKBP12, CaM, and IpTxa have been mapped by cryoEM and 3D difference mapping [8–10,49,50], and in all cases, they bind at least 130 Å away from the ion gate (positions of FKBP12 and Ca^2+^-CaM binding sites are indicated in Figure 4A). We suggest that the conformational changes associated with gating that we have found here are very likely to be the same as the long-range allosteric pathways that convert remote signals sensed through protein/peptide/small molecule–protein interaction in RyR1′s cytoplasmic domains into the appropriate response (e.g., the probability of RyR1′s ion gate to open).

By superimposing the open/closed 3D reconstructions, one can observe regions of density displacement near regions that remain almost stationary. This indicates the presence of structural hinges, i.e., boundaries between regions of RyR1 that move with different breadths. The two more noticeable regions where this takes place are the crevice near domain 4, and the one between domains 5, 9, and 3 (Figure 4A). Interestingly, these hinges correspond to previously mapped binding sites. The crevice near domain 4 is the target for IpTxa and Ca^2+^-CaM [10,50]. Likewise, the intersection between domains 5, 9, and 3, constitutes the FKBP12 binding site [9,10]. Thus, it appears that the hinges may constitute regulatory sites where binding of a relative small effector could produce optimal effect.

### Solving the Controversy of the RyR1 Ion Pathway in the Closed State

It has been previously reported that the dimension of the closed ion gate in a 9.6 Å reconstruction of RyR1 is 15 Å [17]. This is surprising because it is almost twice the size of the pore we have found in our 10.2 and 10.3 Å resolution reconstructions of RyR1 in the closed state and 20% larger than the open ion gate reported here (Figure 11). A pore of 15 Å would leave a large gap that, based on the dimensions of open ion gates for other known cation channels, should not be impermeable to Ca^2+^ ions. The inner branches were not observed in this 9.6 Å 3D reconstruction, and the density in several portions of their putative inner helices is discontinuous (Figure 5B), which raises the possibility that this reconstruction was obtained from a preparation that contained a mixture of open and closed conformations. Such heterogeneity would give a low signal-to-noise ratio in those parts of the structure that change conformation during ion gating. The presence of a low signal-to-noise ratio in their reconstruction required the assistance of helix hunter [51] to identify the putative helices rather than being able to see them directly by increasing the threshold as was done here. There are several portions of their putative inner helices that do not overlap with either our closed- or open-state 3D maps (compare Figure 5B with Figures 3B, 3D, and 5A). Ludtke et al. interpreted their results as meaning that the inner helices of RyR1 in the closed state are more similar to an open than a closed K^+^ channel conformation. This contradicts our report here in which cross-correlation measurements between our open and closed states and all K^+^ channels indicated a direct equivalence of physiological state and inner helix conformation (Figure 6). Finally, the fact that we provide three independent 3D reconstructions supports further the ion gate dimensions and inner helix conformation of open/closed RyR1, and that they are in a similar range of these reported for the K^+^ channels.

### A Heuristic Gating Mechanism for RyR1

Based on our results, we propose that three structures, the inner branches, the inner helices, and h1 densities, by forming a mobile axial structure, are the three main gating effectors. In the closed state, the two right-handed bundles (inner helices, inner branches) form the high-density constriction (ion gate) at their meeting point. In going from the closed to the open state, both sets of bundles relax and appear to contribute equally to lowering the density of the ion gate (see arrows in Figure 3B). The h1 densities also contribute to the constricting effect in the closed state and move outwards as the gate opens. The resulting profile of the pore along the ion pathway looks dramatically different as it transitions from the closed to the open state. Such a three-way mechanism appears to constitute a very efficient mechanism to open and close the ion gate and is compatible with the complex regulation of RyR1 through its interaction with the DHPR and other exogenous or intracellular modulators [52].

### Concluding Remarks

In summary, we have obtained the 3D reconstructions of the hydrated RyR1-FKBP12 complex both in open and closed conformations. The use of neuroactive PCB 95 [18,53] to favor the stability of the full open conformation of the RyR1 channel enabled 3D reconstructions of the ion pathway with high detail. The conformational change of the peripheral cytoplasmic domains is directly related to conformational changes in the transmembrane domain. The architecture of the RyR1 appears to be designed to support precise long-range allosteric pathways such as these involved in efficient coupling with the voltage sensor and in the modulation by ligands such as FKBP12 and CaM. Finally, we have shown that there is a striking similarity between the architectural organization of the transmembrane α helices of the K^+^ channel family and those of RyR1. Beyond this similarity, we find that the inner branches, a structure that connects the cytoplasmic domains of RyR1 to the ion gate, appear to play a direct role in ion gating.

## Materials and Methods

### Materials.

[^3^H]Ryanodine ([^3^H]Ry; 60–90 Ci/mmol; >99% pure) was purchased from Perkin-Elmer New England Nuclear. PCB 95 (>99% pure) was purchased from Ultra Scientific. All other reagents were of the highest purity commercially available.

### RyR1 purification.

RyR1 was purified from rabbit skeletal muscle to concentrations of 2 mg/ml as previously described [7]. Prior to freezing, all RyR1s were incubated with FKBP12 (Sigma) at a molar ratio of 8× for 20–40 min. Final buffer conditions to lock the RyR1 into the closed state were 20 mM Na-MOPS (pH 7.4), 0.9 M NaCl, 0.5% CHAPS, 2 mM DTT, 2 mM EGTA, 5 μg/ml aprotinin, 5 μg/ml leupeptin, and 2.5 μg/ml Pefabloc. To set RyR1 in the open state, the same buffer was used except that 10 μM PCB 95 and 50 μM Ca^2+^ were added and EGTA was excluded.

### Single-channel experiments.

Bilayers were made of phosphatidylethanolamine: phosphatidylserine: phosphatidylcholine (5:3:2 w/w, Avanti Polar Lipids) dissolved in decane at a final concentration of 30 mg/ml. The bilayer partitioned two chambers (*cis* and *trans*) containing buffer solution (in mM) 500 CsCl, 50 μM Ca^2+^, and 20 Hepes-Tris (pH 7.4) on *cis* and 50 CsCl, 7 μM Ca^2+^, and 20 Hepes-Tris (pH 7.4) on *trans*. The addition of protein was made to the *cis* solution that was held at the virtual ground, whereas the *trans* solution was connected to the head stage input of an amplifier (Bilayer Clamp BC 525C; Warner Instruments). Purified RyR1-FKBP12 complexes preincubated for 20–40 min were introduced to *cis* solution. Upon the incorporation of a single RyR1 channel into BLM, the *cis* chamber was perfused with *cis* solution to prevent additional channel incorporation. Single-channel gating was monitored and recorded at a holding potential of −40 mV (applied to the *trans* side). The sidedness (cytosolic) of the channel was verified by the positive response to addition of micromolar Ca^2+^ and response to 2 μM ryanodine and 5 μM Ruthenium Red (at the end of the experiment). The amplified current signals, filtered at 1 kHz (Low-Pass Bessel Filter 8 Pole; Warner Instrument,) were digitized and acquired at a sampling rate of 10 kHz (Digidata 1320A; Axon-Molecular Devices). All the recordings were made for a duration between 12 s and 6 min under each experimental condition. The channel open probability (*P*_o_) was calculated using Clampfit, pClamp software 9.0 (Axon-Molecular Devices) without further filtration.

### [^3^H]Ry binding.

Equilibrium measurements of specific high-affinity [^3^H]Ry binding were determined as previously indicated [20,54]. Junctional SR vesicles of rabbit skeletal muscle (50 μg protein/ml) were incubated with or without PCB 95 in buffer containing 20 mM HEPES (pH 7.4), 250 mM KCl, 15 mM NaCl, defined concentration of CaCl_2_, and 2 nM [^3^H]Ry for 3 h at 37 °C. The reactions were quenched by filtration through GF/B glass fiber filters and washed twice with ice-cold harvest buffer (20 mM Tris-HCl, or 20 mM Hepes, 250 mM KCl, 15 mM NaCl, 50 μM CaCl_2_ [pH7.4]). Nonspecific binding was determined by incubating JSR vesicles with 1,000-fold excess unlabeled ryanodine. Each of the conditions was replicated four times in two separate junctional SR preparations, and each of the readings was performed in triplicate or quadruplicate.

### Cryo-electron microscopy.

A 5-μl aliquot of the 2–4 mg/ml RyR1-FKBP12 complex incubation mixture was adsorbed onto a glow-discharged quantifoil holey grid and the excess blotted off with Whatman 540 filter paper. The sample was vitrified by plunging the grid into liquid ethane. CryoEM was performed on a FEI Tecnai F20 FEG microscope operated at 200 kV. Untilted images with defoci between 2.5 and 4.0 μm were recorded on Kodak SO-163 film under standard low-dose conditions (dose <10 e^−^/Å^2^) at a nominal magnification of 50,000×.

### Single-particle image processing.

A total of 257 and 233 micrographs for the closed and open states, respectively, were digitized on a Zeiss SCAI scanner at a step size of 7 μm, and subsequently binned down to a final pixel size of 2.8 Å. A total of 15,625 and 18,527 particles for the closed and open states, respectively, were selected interactively using the program WEB. The defocus parameters were determined for every particle using CTFTILT [55]. Individual particles were subjected to a reference-based algorithm starting from an initial 3D model of RyR1 [7] filtered to 40 Å resolution where no substructure is detectable, thus avoiding model bias. Fifty percent of the particles from each dataset with the lowest cross-correlation with the 3D model were discarded. This was followed by several iterations of refinement until the shifts and rotations stabilized. The final number of particles was 9,331 and 8,133 particles for the closed and open states, respectively. Reference alignment and 3D reconstruction enforcing 4-fold symmetry were performed using the program FREALIGN [56], which takes account of phase and amplitude contrast transfer function (CTF) correction for every particle. This program has implemented a weighting scheme to correct for noise bias, an artifact that could result in an artificial overestimation of the resolution [57]. Resolution values were calculated according to the Fourier shell correlation (FSC) curve between two half datasets. The 0.143 cutoff [26] was chosen because it was optimistic with respect to the 5 σ noise correction calculated taking into account the 4-fold symmetry (and thus data redundancy) of the RyR1. The final 3D structure of RyR-FKBP12 was normalized and filtered to a resolution of 10.2 Å using a B factor of −300 Å^3^. The mean and standard deviation values of the volume were calculated within a spherical mask of the same diameter as that used in the iterative alignments. For 3D difference mapping, both 3D reconstructions were filtered to 18 Å resolution and normalized by adjusting the average and standard deviation of densities in both reconstructions to the same level as previously done [9]. Then the open-state RyR1 3D reconstruction was directly subtracted from the closed-state RyR1 3D reconstruction and vice versa. No further data manipulation such as postsubtraction filtering or masking was performed. SPIDER software [58] was used for preparation of the initial volumes, normalization, 3D difference mapping, filtration of the Protein Data Bank (pdb) files for comparison with the cryoEM density maps, and calculation of cross-correlation values. Image rendering, docking of atomic structures, and alignment of the other RyR1 3D reconstruction from the database were performed in Chimera [59] (http://www.ebi.ac.uk/pdbe/emdb/). Both closed-state RyR1 3D reconstructions that have been previously published [7,17] are available in the Electron Microscopy Database (http://www.ebi.ac.uk/msd-srv/docs/emdb/).

### Secondary structure prediction.

Hydropathicity, transmembrane propensity, and α helical prediction analyses were performed using several packages available on public servers. The different packages for α helical prediction provided reasonable overlapping sequence segments. The proposed secondary structure is based on the PSIPRED prediction method [60] (http://bioinf.cs.ucl.ac.uk/psipred/).

### Accession numbers.

Electron Microscopy Data Bank (http://www.ebi.ac.uk/pdbe/emdb/) accession numbers for the structures of the RyR1 in closed and open conformations are 1606 and 1607 respectively.

## Supporting Information

Video S1 Conformational Changes of RyR1 upon Gating (1) The RyR1 alternates between its open and closed conformations while moving. (2) A vertical plane cuts through the side view until the 4-fold axis, the threshold is increased, and the transmembrane domain is magnified. (3) The RyR1 alternates between its open and closed states.
(1.80 MB MOV)

Video S2 Gating of RyR1 along the Ion Pathway The viewer's perspective moves along the 4-fold axis of the RyR1, going from the cytoplasmic domains towards the SR lumen. The conformational changes are shown for the cytosolic constriction, the inner branches, the ion gate, the inner helices, and the pore helices.
(3.58 MB MOV)

## Abbreviations

BLM: bilayer lipid membrane
CaM: calmodulin
cryoEM: cryo-electron microscopy
DHPR: dihydropyridine receptor
FKBP12: FK506-binding protein 12 kDa
IpTxA: imperatoxin A
nAChR: nicotinic acetylcholine receptor
RyR: ryanodine receptor
SR: sarcoplasmic reticulum

## References

[pbio-1000085-b001] Brini M, Carafoli E (2000). Calcium signalling: a historical account, recent developments and future perspectives. Cell Mol Life Sci.

[pbio-1000085-b002] Nakai J, Dirksen RT, Nguyen HT, Pessah IN, Beam KG (1996). Enhanced dihydropyridine receptor channel activity in the presence of ryanodine receptor. Nature.

[pbio-1000085-b003] Serysheva II, Orlova EV, Chiu W, Sherman MB, Hamilton SL (1995). Electron cryomicroscopy and angular reconstitution used to visualize the skeletal muscle calcium release channel. Nat Struct Biol.

[pbio-1000085-b004] Radermacher M, Rao V, Grassucci R, Frank J, Timerman AP (1994). Cryo-electron microscopy and three-dimensional reconstruction of the calcium release channel/ryanodine receptor from skeletal muscle. J Cell Biol.

[pbio-1000085-b005] Wagenknecht T, Samso M (2002). Three-dimensional reconstruction of ryanodine receptors. Front Biosci.

[pbio-1000085-b006] Samso M, Wagenknecht T (1998). Contributions of electron microscopy and single-particle techniques to the determination of the ryanodine receptor three-dimensional structure. J Struct Biol.

[pbio-1000085-b007] Samso M, Wagenknecht T, Allen PD (2005). Internal structure and visualization of transmembrane domains of the RyR1 calcium release channel by cryo-EM. Nat Struct Mol Biol.

[pbio-1000085-b008] Samso M, Wagenknecht T (2002). Apocalmodulin and Ca2+-calmodulin bind to neighboring locations on the ryanodine receptor. J Biol Chem.

[pbio-1000085-b009] Samso M, Shen X, Allen PD (2006). Structural characterization of the RyR1-FKBP12 interaction. J Mol Biol.

[pbio-1000085-b010] Wagenknecht T, Radermacher M, Grassucci R, Berkowitz J, Xin HB (1997). Locations of calmodulin and FK506-binding protein on the three-dimensional architecture of the skeletal muscle ryanodine receptor. J Biol Chem.

[pbio-1000085-b011] Orlova EV, Serysheva II, van Heel M, Hamilton SL, Chiu W (1996). Two structural configurations of the skeletal muscle calcium release channel. Nat Struct Biol.

[pbio-1000085-b012] Serysheva II, Schatz M, van Heel M, Chiu W, Hamilton SL (1999). Structure of the skeletal muscle calcium release channel activated with Ca2+ and AMP-PCP. Biophys J.

[pbio-1000085-b013] Alam A, Jiang Y (2009). High-resolution structure of the open NaK channel. Nat Struct Mol Biol.

[pbio-1000085-b014] Miyazawa A, Fujiyoshi Y, Unwin N (2003). Structure and gating mechanism of the acetylcholine receptor pore. Nature.

[pbio-1000085-b015] Doyle DA, Morais Cabral J, Pfuetzner RA, Kuo A, Gulbis JM (1998). The structure of the potassium channel: molecular basis of K+ conduction and selectivity. Science.

[pbio-1000085-b016] Kuo A, Gulbis JM, Antcliff JF, Rahman T, Lowe ED (2003). Crystal structure of the potassium channel KirBac1.1 in the closed state. Science.

[pbio-1000085-b017] Ludtke SJ, Serysheva II, Hamilton SL, Chiu W (2005). The pore structure of the closed RyR1 channel. Structure (Camb).

[pbio-1000085-b018] Wong PW, Brackney WR, Pessah IN (1997). Ortho-substituted polychlorinated biphenyls alter microsomal calcium transport by direct interaction with ryanodine receptors of mammalian brain. J Biol Chem.

[pbio-1000085-b019] Wong PW, Pessah IN (1996). Ortho-substituted polychlorinated biphenyls alter calcium regulation by a ryanodine receptor-mediated mechanism: structural specificity toward skeletal- and cardiac-type microsomal calcium release channels. Mol Pharmacol.

[pbio-1000085-b020] Pessah IN, Stambuk RA, Casida JE (1987). Ca2+-activated ryanodine binding: mechanisms of sensitivity and intensity modulation by Mg2+, caffeine, and adenine nucleotides. Mol Pharmacol.

[pbio-1000085-b021] Hille B (2001). Ion channels of excitable membranes. 3rd edition.

[pbio-1000085-b022] Wong PW, Pessah IN (1997). Noncoplanar PCB 95 alters microsomal calcium transport by an immunophilin FKBP12-dependent mechanism. Mol Pharmacol.

[pbio-1000085-b023] Timerman AP, Ogunbumni E, Freund E, Wiederrecht G, Marks AR (1993). The calcium release channel of sarcoplasmic reticulum is modulated by FK-506-binding protein. Dissociation and reconstitution of FKBP-12 to the calcium release channel of skeletal muscle sarcoplasmic reticulum. J Biol Chem.

[pbio-1000085-b024] Mayrleitner M, Timerman AP, Wiederrecht G, Fleischer S (1994). The calcium release channel of sarcoplasmic reticulum is modulated by FK-506 binding protein: effect of FKBP-12 on single channel activity of the skeletal muscle ryanodine receptor. Cell Calcium.

[pbio-1000085-b025] Glaeser RM, Tong L, Kim SH (1989). Three-dimensional reconstructions from incomplete data: interpretability of density maps at “atomic” resolution. Ultramicroscopy.

[pbio-1000085-b026] Rosenthal PB, Henderson R (2003). Optimal determination of particle orientation, absolute hand, and contrast loss in single-particle electron cryomicroscopy. J Mol Biol.

[pbio-1000085-b027] Long SB, Tao X, Campbell EB, MacKinnon R (2007). Atomic structure of a voltage-dependent K+ channel in a lipid membrane-like environment. Nature.

[pbio-1000085-b028] Jiang Y, Lee A, Chen J, Cadene M, Chait BT (2002). The open pore conformation of potassium channels. Nature.

[pbio-1000085-b029] Jiang Y, Lee A, Chen J, Ruta V, Cadene M (2003). X-ray structure of a voltage-dependent K+ channel. Nature.

[pbio-1000085-b030] Fujiyoshi Y (1998). The structural study of membrane proteins by electron crystallography. Adv Biophys.

[pbio-1000085-b031] Unwin N (1993). Nicotinic acetylcholine receptor at 9 A resolution. J Mol Biol.

[pbio-1000085-b032] Chiu W, Baker ML, Jiang W, Dougherty M, Schmid MF (2005). Electron cryomicroscopy of biological machines at subnanometer resolution. Structure (Camb).

[pbio-1000085-b033] Jiang Y, Lee A, Chen J, Cadene M, Chait BT (2002). Crystal structure and mechanism of a calcium-gated potassium channel. Nature.

[pbio-1000085-b034] Lee SY, Lee A, Chen J, MacKinnon R (2005). Structure of the KvAP voltage-dependent K+ channel and its dependence on the lipid membrane. Proc Natl Acad Sci U S A.

[pbio-1000085-b035] Long SB, Campbell EB, Mackinnon R (2005). Crystal structure of a mammalian voltage-dependent Shaker family K+ channel. Science.

[pbio-1000085-b036] Welch W, Rheault S, West DJ, Williams AJ (2004). A model of the putative pore region of the cardiac ryanodine receptor channel. Biophys J.

[pbio-1000085-b037] Zorzato F, Fujii J, Otsu K, Phillips M, Green NM (1990). Molecular cloning of cDNA encoding human and rabbit forms of the Ca2+ release channel (ryanodine receptor) of skeletal muscle sarcoplasmic reticulum. J Biol Chem.

[pbio-1000085-b038] Du GG, Sandhu B, Khanna VK, Guo XH, MacLennan DH (2002). Topology of the Ca2+ release channel of skeletal muscle sarcoplasmic reticulum (RyR1). Proc Natl Acad Sci U S A.

[pbio-1000085-b039] Williams AJ, West DJ, Sitsapesan R (2001). Light at the end of the Ca(2+)-release channel tunnel: structures and mechanisms involved in ion translocation in ryanodine receptor channels. Q Rev Biophys.

[pbio-1000085-b040] Schug ZT, da Fonseca PC, Bhanumathy CD, Wagner L, Zhang X (2008). Molecular characterization of the Inositol 1,4,5-trisphosphate receptor pore-forming segment. J Biol Chem.

[pbio-1000085-b041] Xu L, Wang Y, Gillespie D, Meissner G (2006). Two rings of negative charges in the cytosolic vestibule of type-1 ryanodine receptor modulate ion fluxes. Biophys J.

[pbio-1000085-b042] Unwin N (2005). Refined structure of the nicotinic acetylcholine receptor at 4A resolution. J Mol Biol.

[pbio-1000085-b043] Lee EH, Allen PD (2007). Homo-dimerization of RyR1 C-terminus via charged residues in random coils or in an alpha-helix. Exp Mol Med.

[pbio-1000085-b044] Cortes DM, Cuello LG, Perozo E (2001). Molecular architecture of full-length KcsA: role of cytoplasmic domains in ion permeation and activation gating. J Gen Physiol.

[pbio-1000085-b045] Balshaw D, Gao L, Meissner G (1999). Luminal loop of the ryanodine receptor: a pore-forming segment. Proc Natl Acad Sci U S A.

[pbio-1000085-b046] Zhao M, Li P, Li X, Zhang L, Winkfein RJ (1999). Molecular identification of the ryanodine receptor pore-forming segment. J Biol Chem.

[pbio-1000085-b047] Du GG, Guo X, Khanna VK, MacLennan DH (2001). Functional characterization of mutants in the predicted pore region of the rabbit cardiac muscle Ca(2+) release channel (ryanodine receptor isoform 2). J Biol Chem.

[pbio-1000085-b048] Lynch PJ, Tong J, Lehane M, Mallet A, Giblin L (1999). A mutation in the transmembrane/luminal domain of the ryanodine receptor is associated with abnormal Ca2+ release channel function and severe central core disease. Proc Natl Acad Sci U S A.

[pbio-1000085-b049] Wagenknecht T, Grassucci R, Berkowitz J, Wiederrecht GJ, Xin HB (1996). Cryoelectron microscopy resolves FK506-binding protein sites on the skeletal muscle ryanodine receptor. Biophys J.

[pbio-1000085-b050] Samso M, Trujillo R, Gurrola GB, Valdivia HH, Wagenknecht T (1999). Three-dimensional location of the imperatoxin A binding site on the ryanodine receptor. J Cell Biol.

[pbio-1000085-b051] Jiang W, Baker ML, Ludtke SJ, Chiu W (2001). Bridging the information gap: computational tools for intermediate resolution structure interpretation. J Mol Biol.

[pbio-1000085-b052] Fill M, Copello JA (2002). Ryanodine receptor calcium release channels. Physiol Rev.

[pbio-1000085-b053] Kenet T, Froemke RC, Schreiner CE, Pessah IN, Merzenich MM (2007). Perinatal exposure to a noncoplanar polychlorinated biphenyl alters tonotopy, receptive fields, and plasticity in rat primary auditory cortex. Proc Natl Acad Sci U S A.

[pbio-1000085-b054] Pessah IN, Waterhouse AL, Casida JE (1985). The calcium-ryanodine receptor complex of skeletal and cardiac muscle. Biochem Biophys Res Commun.

[pbio-1000085-b055] Mindell JA, Grigorieff N (2003). Accurate determination of local defocus and specimen tilt in electron microscopy. J Struct Biol.

[pbio-1000085-b056] Grigorieff N (2007). FREALIGN: high-resolution refinement of single particle structures. J Struct Biol.

[pbio-1000085-b057] Stewart A, Grigorieff N (2004). Noise bias in the refinement of structures derived from single particles. Ultramicroscopy.

[pbio-1000085-b058] Frank J, Radermacher M, Penczek P, Zhu J, Li Y (1996). SPIDER and WEB: processing and visualization of images in 3D electron microscopy and related fields. J Struct Biol.

[pbio-1000085-b059] Huang CC, Couch GS, Pettersen EF, Ferrin TE (1996). Chimera: an extensive molecular modeling application constructed using standard components. Pac Symp Biocomput.

[pbio-1000085-b060] Bryson K, McGuffin LJ, Marsden RL, Ward JJ, Sodhi JS (2005). Protein structure prediction servers at University College London. Nucleic Acids Res.

